# Cognitive Impairment in Tuberculous Meningitis

**DOI:** 10.1093/cid/ciac831

**Published:** 2022-10-20

**Authors:** Angharad G Davis, Anna J Dreyer, Christine Albertyn, Mpumi Maxebengula, Cari Stek, Sean Wasserman, Suzaan Marais, Kathleen Bateman, Mark Solms, John Joska, Robert J Wilkinson, Sam Nightingale

**Affiliations:** The Francis Crick Institute, London, United Kingdom; Faculty of Life Sciences, University College London, London, United Kingdom; Wellcome Centre for Infectious Diseases Research in Africa and Institute of Infectious Disease and Molecular Medicine, University of Cape Town, Cape Town, Republic of South Africa; Division of Neuropsychiatry, Department of Psychiatry and Mental Health, HIV Mental Health Research Unit, Neuroscience Institute, University of Cape Town, Cape Town, South Africa; Division of Neurology, Department of Medicine, Stellenbosch University, Cape Town, South Africa; Division of Neurology, Department of Medicine, University of Cape Town, Cape Town, South Africa; Wellcome Centre for Infectious Diseases Research in Africa and Institute of Infectious Disease and Molecular Medicine, University of Cape Town, Cape Town, Republic of South Africa; Wellcome Centre for Infectious Diseases Research in Africa and Institute of Infectious Disease and Molecular Medicine, University of Cape Town, Cape Town, Republic of South Africa; Department of Infectious Diseases, Imperial College London, London, United Kingdom; Wellcome Centre for Infectious Diseases Research in Africa and Institute of Infectious Disease and Molecular Medicine, University of Cape Town, Cape Town, Republic of South Africa; Division of Infectious Diseases and HIV Medicine, Department of Medicine, University of Cape Town, Cape Town, South Africa; Division of Neurology, Department of Medicine, University of Cape Town, Cape Town, South Africa; Neurology Research Group, Neuroscience Institute, University of Cape Town, Cape Town, South Africa; Division of Neurology, Department of Medicine, University of Cape Town, Cape Town, South Africa; Division of Psychology, University of Cape Town, Cape Town, South Africa; Division of Neuropsychiatry, Department of Psychiatry and Mental Health, HIV Mental Health Research Unit, Neuroscience Institute, University of Cape Town, Cape Town, South Africa; The Francis Crick Institute, London, United Kingdom; Faculty of Life Sciences, University College London, London, United Kingdom; Wellcome Centre for Infectious Diseases Research in Africa and Institute of Infectious Disease and Molecular Medicine, University of Cape Town, Cape Town, Republic of South Africa; Department of Infectious Diseases, Imperial College London, London, United Kingdom; Division of Neuropsychiatry, Department of Psychiatry and Mental Health, HIV Mental Health Research Unit, Neuroscience Institute, University of Cape Town, Cape Town, South Africa

**Keywords:** tuberculous meningitis, HIV, cognitive impairment, functional impairment, treatment adherence

## Abstract

**Background:**

Cognitive impairment is reported as a common complication in adult tuberculous meningitis (TBM), yet few studies have systematically assessed the frequency and nature of impairment. Moreover, the impact of impairment on functioning and medication adherence has not been described.

**Methods:**

A cognitive test battery (10 measures assessing 7 cognitive domains) was administered to 34 participants with human immunodeficiency virus (HIV)–associated TBM 6 months after diagnosis. Cognitive performance was compared with that a comparator group of 66 people with HIV without a history of tuberculosis. A secondary comparison was made between participants with TBM and 26 participants with HIV 6 months after diagnosis of tuberculosis outside the central nervous system (CNS). Impact on functioning was evaluated, including through assessment of medication adherence.

**Results:**

Of 34 participants with TBM, 16 (47%) had low performance on cognitive testing. Cognition was impaired across all domains. Global cognitive performance was significantly lower in participants with TBM than in people with HIV (mean T score, 41 vs 48, respectively; *P <* .001). These participants also had lower global cognition scores than those with non-CNS tuberculosis (mean global T score, 41 vs 46; *P* = .02). Functional outcomes were not significantly correlated with cognitive performance in the subgroup of participants in whom this was assessed (n = 19).

**Conclusions:**

Low cognitive performance following HIV-associated TBM is common. This effect is independent of, and additional to, effects of HIV and non-CNS tuberculosis disease. Further studies are needed to understand longer-term outcomes, clarify the association with treatment adherence, a key predictor of outcome in TBM, and develop context-specific tools to identify individuals with cognitive difficulties in order to improve outcomes in TBM.

## Teaser

Cognitive impairment following tuberculous meningitis is poorly described. In a case control study we demonstrate that low cognitive performance affects ∼50% of patients at 6 months following diagnosis HIV-associated TBM, and is significantly worse than that seen in HIV alone

Tuberculous meningitis (TBM) affects approximately 100 000 people per year worldwide [1]. Cognitive impairment can occur in TBM, but only 4 studies have reported its frequency. Two of the studies used brief screening tests to assess cognition [2, 3], and another gathered information on cognition from clinical history alone [4]. The only study to undertake more comprehensive cognitive testing assessed a small group of 17 participants with TBM [5]. No studies have been undertaken in an African setting where TBM and human immunodeficiency virus (HIV) are endemic.

Better understanding of cognitive impairment in TBM is crucial for several reasons. First, objective measures of cognition are quantitative measures of outcome that are infrequently used in clinical trials in adult TBM. By contrast, pediatric studies routinely consider these as part of a neurodevelopmental assessment [6]. Developing an accessible battery of cognitive tests that assesses cognitive domains known to be impaired in TBM would improve the precision of measurable outcomes for TBM studies. Second, recent TBM pathogenesis studies have unveiled mechanisms of brain injury, such as the up-regulation of neuroexcitatory pathways [7] and release of damage-associated proteins also seen in neurodegenerative conditions [8].

These findings encourage us to better understand whether cognitive impairment leads to longer-term disability in TBM, in particular whether cognitive impairment is focal and attributable to discrete structural abnormalities in the brain (eg, stroke or tuberculomas) and/or whether there is a clinical presentation in keeping with a diffuse cortical or subcortical process at play. Most importantly, understanding cognitive and functional impairment in TBM, particularly its effect on treatment adherence, will improve the long-term care of patients, including the provision of appropriate resources in recovery following TBM.

In a case-control study of HIV-associated TBM (referred to hereafter as HIV-TBM) we aimed to do the following: (1) evaluate the frequency of cognitive impairment in HIV-TBM using formal cognitive testing alongside physician assessment; (2) assess the pattern of impairment, and correlations with radiological and neurological measures, to understand whether cognitive impairment relates to focal brain injury and/or diffuse inflammation; (3) assess the suitability of currently available screening tools to identify cognitive impairment; and (4) measure the impact of impaired cognitive performance on functional outcomes, including treatment adherence.

## METHODS

### Participants

We drew participants from 3 parent studies outlined in Table 1. All studies were performed in similar populations in a low-income, periurban area of Cape Town, South Africa, with high HIV and tuberculosis prevalence. Participants formed 3 groups: (1) those with HIV-TBM (from the LASER-TBM and Albertyn studies); comparator group 1, including people with HIV (PWH) with no history of tuberculosis (from the CONNECT study); and (3) comparator group 2, including PWH who had non–central nervous system (CNS) tuberculosis (from the Albertyn study).

**Table 1.** Parent Studies

Study Characteristic	LASER-TBM Study	Albertyn Study	CONNECT Study
Study design	Phase 2a randomized, open-label clinical trial of intensified antibiotics and high-dose aspirin in HIV-TBM [9]	Prospective case-control study evaluating cognitive and functional impairment in HIV-TBM (control group, other forms of tuberculosis [non-CNS] )	Prospective case-control study evaluating cognition, neuropsychiatric symptoms, and neuroinflammation in PWH switching from efavirenz to dolutegravir
Ethical approval no.	UCT HREC 293/2018	UCT HREC 565/2014	UCT HREC 017/2019
Participants recruited, no.	52	27 With TBM and 25 controls (non-CNS tuberculosis)	180 HIV-positive participants and 60 HIV-negative controls
Setting	Inpatient	Inpatient	Outpatient
Inclusion criteria	Adults with TBM (definite, probable, or possible); confirmed diagnosis of HIV	Adults with TBM (definite or probable); confirmed diagnosis of HIV	180 PWH studied before and after switch from efavirenz to dolutegravir
Exclusion criteria	Many exclusions related to RCT (see [9]); none related to prior head injury, neurological disease, or drug/alcohol dependance	Significant prior CNS disease (stroke, opportunistic CNS infection, significant head injury, or dementia); active alcohol or substance abuse/dependence; poor socioeconomic support	Excessive drug or alcohol misuse; history of CNS infection (including meningitis); previous stroke; major head injury (loss of consciousness for >30 min)
Follow-up time point	6 mo	6 mo	Baseline and after switch assessments (6–12 mo)

Abbreviations: CNS, central nervous system; HIV, human immunodeficiency virus; HIV-TBM, HIV-associated tuberculous meningitis; HREC, Human Research Ethical Committee; PWH, people with HIV; RCT, randomized controlled trial; TBM, tuberculous meningitis; UCT, University of Cape Town.

Normative cognitive data were drawn from HIV-negative individuals selected to be demographically similar to PWH within CONNECT (described in Statistical Analysis).

The studies were approved by the University of Cape Town's Faculty of Health Sciences Research Ethical Committee (Table 1). The sample size was pragmatic, combining 2 unpublished data sets of patients with HIV-TBM and patients with non-CNS tuberculosis (comparator group 2). The inclusion of PWH (comparator group 1) at a 1:2 ratio was done to increase statistical power.

### Outcome Measures and Procedure

Assessment took place at 6 months after the diagnosis of TBM or non-CNS tuberculosis or at the time of enrollment in comparator group 1, as dictated by the parent studies.

### Baseline Assessments

We graded the severity of TBM at baseline using the modified British Medical Research Council scale [10] and classified TBM as “definite,” “probable,” or “possible” [11]. We collected data on education, drug and alcohol use; in the LASER and CONNECT studies, the Alcohol Use Disorders Identification Test (AUDIT) [12] (cutoff defining high risk, ≥20) and the Drug Use Disorders Identification Test (DUDIT) [13] (cutoff, ≥6 for men and ≥ 2 for women) questionnaires were used to ascertain alcohol and drug use, respectively. Within the Albertyn cohort, active alcohol or illicit drug use was an exclusion criterion.

### Cognitive Testing

Cognitive testing was performed in the participant’s first language. An identical cognitive test battery was administered across the 3 studies to assess 10 measures in 7 cognitive domains (Supplementary Box 1), based on a battery widely used in South Africa [14]. Participants in the LASER-TBM study also underwent a comprehensive neurological examination to identify focal cortical syndromes (Supplementary Box 2).

### Imaging

Within the LASER cohort we correlated results from cognitive assessment with brain computed tomography scans and/or magnetic resonance imaging (≤2 months since diagnosis) reported by an independent blinded neuroradiologist.

### Mental Health Assessment

All participants completed a Beck Depression Inventory [15] or the CES-D [16] (to assess mood (cutoff score for “depression,” >17 and ≥16, respectively).

### Functional Measures

We administered the Patient's Assessment of Own Functioning Inventory (PAOFI) [17] in all participants recruited to the LASER-TBM study. We calculated the total score and the total number of responses with “affirmative responses,” as described elsewhere [18, 19]. Lower total PAOFI scores indicate higher levels of functioning; ≥ 3 affirmative responses indicate “functional impairment.” Within the LASER-TBM cohort we evaluated treatment adherence for the first 56 days of treatment. Self-reported adherence was assessed by asking participants at each visit if they had missed any doses since their last visit. Observed adherence was assessed by totaling the number of missed doses noted on the pill count at each study visit.

### Cognitive Screening Measures

Two screening measures—the Montreal cognitive assessment (MoCA) [20] (cutoff score indicating impairment, ≤26) and CAT-Rapid version 2.0 [21] (cutoff score indicating impairment, ≤16)—were used in LASER-TBM participants.

### Statistical Analysis

**Figure 1.**
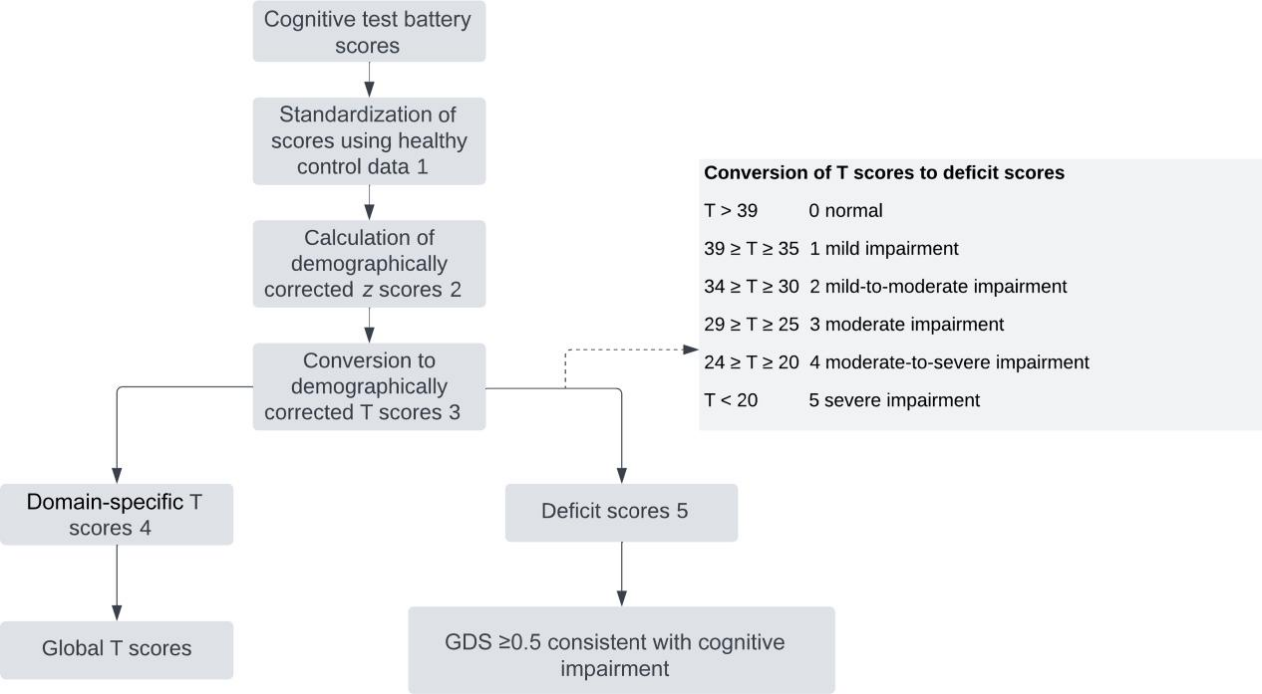
Processing of cognitive test battery data. First, standardization of scores used normative scores calculated from healthy control data collected by the CONNECT study. These data were collected between 2018 and 2020 from healthy human immunodeficiency virus (HIV)–negative community-dwelling individuals who presented to the same community health clinics in Gugulethu from which the people with HIV (PWH) comparator group was recruited, within the same area of Cape Town where participants with tuberculous meningitis (TBM) and tuberculosis were recruited. The groups therefore shared key demographics (age, ethnicity, language, and education), as well as psychosocial and socioeconomic characteristics. Demographically corrected *z* scores (mean [standard deviation (SD)], 0 [1]) were then calculated using standard regression-based norming processes. The *z* scores were then converted to demographically corrected T scores (mean [SD], 50 [10]). If participants had *z* scores >5 SDs below the mean, the conversion to a T score resulted in a negative T score. In these cases, we assigned a score of 0, the lowest possible T score, to maintain the clinical significance of the low performance. Next, cognitive performance data were summarized into domain-specific and global T scores by taking the average of T scores within each domain and then across domain T scores. T scores were then converted to deficit scores. The overall global deficit score (GDS) was calculated by averaging deficit scores. A cutoff GDS of ≥0.5 has been considered consistent with “cognitive impairment” on cognitive test performance [22]; for the purposes of this study, we termed this group as having “low performance on cognitive testing” while the clinical significance and functional impact in TBM is further explored, aligned with recent trends in the HIV literature [23].

We used R (version 4.1.2; 1 November 2021), RStudio (version 2021.09.0), and GraphPad Prism (version 9.3.1) software to complete all analyses, with the threshold for statistical significance set at α = .05. First, we processed and standardized the cognitive test battery data. This process is outlined in Figure 1, including use of a healthy control group to standardize data and calculation of *z* scores, T scores, global T and global deficit scores (GDSs). Second, we used the Matchit package in R (version 4.1.2; 1 November 2021) software to select comparator group 1 from the parent study CONNECT at a ratio of 1:2 with matching of covariates (age, sex, education) to participants with TBM so that distributions of covariates in the 2 groups were approximately equal. The cognitive test data for this group were subsequently processed as shown in Figure 1.

We checked the normality of data distribution with a Shapiro-Wilk test. We determined between-group differences in baseline and clinical variables using parametric *t* or Mann-Whitney tests (for continuous variables where data were normally or not normally distributed, respectively) or χ^2^ tests (for dichotomous variables). We performed a primary comparison of cognitive test performance between HIV-TBM case patients and comparator group 1 and subsequently between HIV-TBM case patients and comparator group 2.

Using a Mann-Whitney test of significance, we compared total PAOFI scores between participants with HIV-TBM with low cognitive performance and those without. We also compared adherence between these 2 groups by (1) self-report of missing ≥1 dose (compared using Fisher exact test (2) observed adherence from number of missed doses on pill count within the first 56 days of treatment (compared using Mann-Whitney test). We used a 2 × 2 table of agreement to create a Cohen κ value for agreement between the screening tests (CatRAPID cutoff, ≤16; MoCA cutoff ≥26) and a GDS of ≥0.5 on the cognitive test battery and assigned levels of agreement per a published scale [24].

## RESULTS

**Figure 2.**
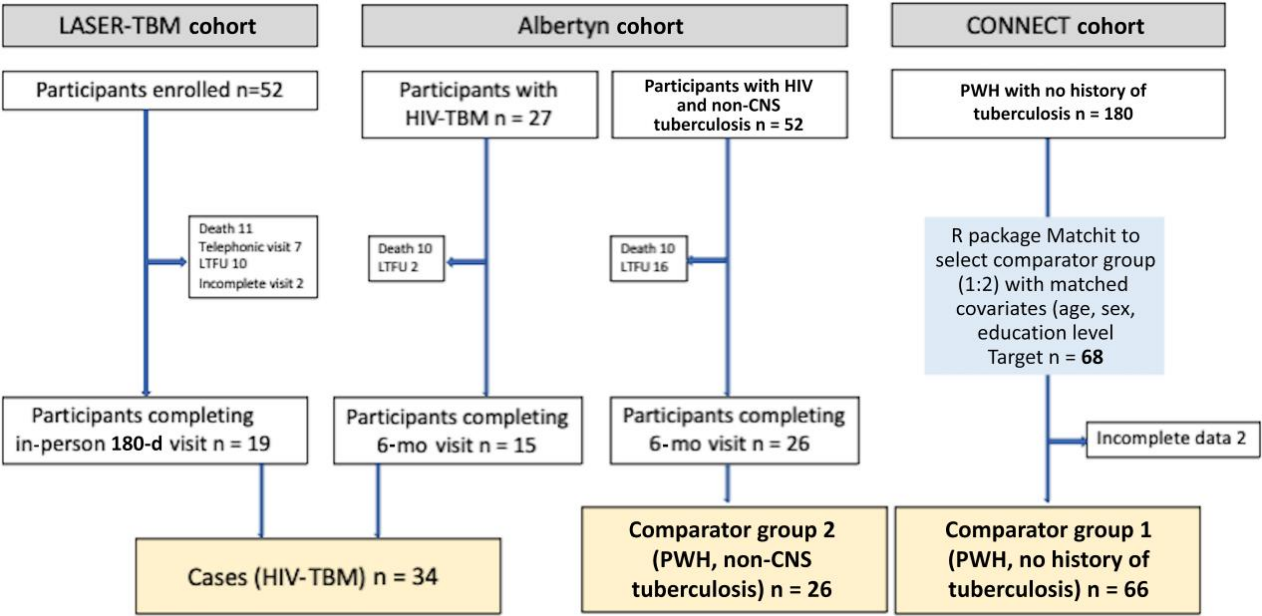
Consort diagram describing enrollments across 3 parent studies. Abbreviations: CNS, central nervous system; HIV, human immunodeficiency virus; HIV-TBM, HIV-associated tuberculous meningitis; LTFU, lost to follow-up; PWH, people with HIV.

We included 34 participants with HIV-TBM (case patients), 66 PWH with no history of tuberculosis (comparator group 1), and 26 PWH with non-CNS tuberculosis (comparator group 2) (Figure 2). Demographics were similar across groups (Table 2). Within the LASER-TBM study, 2 of 19 participants reported head injury resulting in loss of consciousness, 2 participants had “high-risk” alcohol use, and none of the participants had “high-risk” drug use. These were exclusions in the CONNECT and Albertyn studies.

**Table 2.** Baseline Demographic and Key Clinical Characteristics

Characteristic	HIV-TBM (n = 34)	PWH (n = 66)	Non-CNS Tuberculosis (n = 26)
Age, mean (SD), y	36.4 (8.9)	37.4 (7.5)	35.3 (8.0)
Sex, % male	47	41	58
Length of education, mean (SD), y	9.6 (3.1)	10.4 (1.5)	10.2 (1.6)
First language spoken, no. (%)			
ȃisiXhosa	34 (100)	59 (89)	26 (100)
ȃEnglish	0	2 (3)	0
ȃShona	0	3 (5)	0
ȃMissing data	0	2 (3)	0
Baseline BMRC grade			
ȃ1	18 (53)	NA	NA
ȃ2	16 (47)	NA	NA
ȃ3	0	NA	NA
Uniform case definition TBM category			
ȃPossible	7 (21)	NA	NA
ȃProbable	13 (38)	NA	NA
ȃDefinite	14 (41)	NA	NA
HIV details available, no.	34^a^	41^b^	26^a^
CD4 cell count, mean, (SD), cells/μL	177 (183)	533 (293)	88.6 (90.4)

Abbreviations: BMRC, British Medical Research Council; CNS, central nervous system; HIV, human immunodeficiency virus; HIV-TBM, HIV-associated tuberculous meningitis; NA, not applicable; PWH, people with HIV; SD, standard deviation; TBM; tuberculous meningitis.

Details collected at the time of diagnosis of TBM or non-CNS tuberculosis (ie, 6 months before cognitive testing).

Details collected at the time of cognitive testing.

Among participants with HIV-TBM, 16 of 34 (47%) had low performance on cognitive testing consistent with cognitive impairment (GDS ≥0.5). Compared with comparator group 1 (PWH with no history of tuberculosis), those with TBM had worse global T scores (mean score, 41 vs 48, respectively; *P <* .001), with a larger proportion of those in the TBM group meeting the GDS cutoff of ≥0.5 (16 of 34 [47%] vs 17 of 66 [26%)] for comparator group 1; *P* = .03). Domain-specific T scores for all cognitive domains were significantly worse in the TBM group, with the exception of attention and working memory (Table 3). Global T scores were also worse in participants with TBM than in comparator group 2 (PWH with non-CNS tuberculosis) (mean score 41 vs 46, respectively; *P* = .02).

**Table 3.** Domain-Specific T Scores and Global T Scores: Comparison With Comparator Group 1

Score	Score, Mean (SD)^a^		*P* Value
	HIV-TBM (n = 34)	Comparator Group 1^b^ (n = 66)	
Domain T score			
ȃMotor skills	38 (14)	46 (11)	<.05^c^
ȃProcessing speed	36 (15)	47 (7)	<.001^d^
ȃAttention and working memory	47 (10)	49 (9)	.24
ȃFluency	45 (10)	49 (7)	.03^c^
ȃAudioverbal learning and memory	39 (15)	49 (9)	<.001^d^
ȃVisuospatial learning and memory	40 (8)	48 (10)	<.001^d^
ȃExecutive function	41 (14)	47 (10)	.04^c^
Global T score	41 (9)	48 (6)	<.001^d^
GDS suggesting cognitive impairment, no. (%)	16 (47)	17 (26)	.03

Abbreviations: GDS, global deficit score; HIV-TBM, human immunodeficiency virus (HIV)–associated tuberculous meningitis; SD, standard deviation.

Values represent mean (SD) score unless otherwise specified.

Comparator group 1 included people with HIV with no history of tuberculosis.

*P* < .05.

*P* < .001.

**Figure 3.**
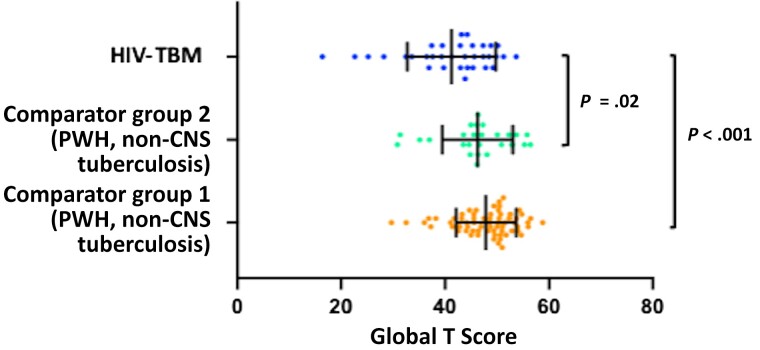
Global T scores. Scatterplot graph displays mean scores with standard deviations, as well as individual values plotted for participants with human immunodeficiency virus (HIV)–associated tuberculous meningitis (HIV-TBM), people with HIV (PWH) with non–central nervous system (CNS) tuberculosis (comparator group 2), and PWH with HIV only (no history of tuberculosis; comparator group 1).

Although a larger proportion of participants with TBM met the GDS cutoff for cognitive impairment (16 of 34 [47%] in HIV-TBM vs 8 of 26 [31%] in comparator group 2), this difference was not statistically significant (*P* = .20) (Table 4). Global T scores were worst in those with HIV-TBM (case patients), better in those with HIV and non-CNS tuberculosis (comparator group 2), and best in those with HIV alone (comparator group 1) (Figure 3). Radiological assessment was performed in 16 of 19 LASER-TBM participants who underwent imaging either as part of the study or for a clinical indication at presentation. Of those 16 participants, 7 (44%) had abnormal imaging findings; all 7 (44%) had meningeal enhancement and 4 had stroke (25%). One participant had multiple calcified granulomas, and another had hydrocephalus. Of the 4 participants with stroke, 3 had a GDS consistent with low cognitive performance. In these participants the profile of abnormality could not be explained by the anatomic location alone. Although in these cases the infarcts likely contributed to the burden of impairment, the scores demonstrated a more global picture. Physician assessment did not reveal any correlation between focal motor or sensory deficits and impairment in a discrete corresponding cognitive domain (eg, owing to stroke or tuberculomas).

**Table 4.** Domain-Specific T Scores and Global T Scores: Comparison With Comparator Group 2

Score	Score, Mean (SD)^a^		*P* Value
	HIV-TBM (n = 34)	Comparator Group 2^b^ (n = 26)	
Domain T score			
ȃMotor skills	38 (14)	44 (8)	.11
ȃProcessing speed	36 (15)	43 (9)	.10
ȃAttention and working memory	47 (10)	52 (9)	.07
ȃFluency	45 (10)	48 (9)	.20
ȃAudioverbal learning and memory	39 (15)	43 (15)	.27
ȃVisuospatial learning and memory	40 (8)	46 (12)	.04^c^
ȃExecutive function	41 (14)	48 (10)	.16
Global T score	41 (9)	46 (7)	.02^c^
GDS suggesting cognitive impairment, no. (%)	16 (47)	8 (31)	.20

Abbreviations: GDS, global deficit score; HIV-TBM, human immunodeficiency virus (HIV)–associated tuberculous meningitis; SD, standard deviation.

Values represent mean (SD) score unless otherwise specified.

Comparator group 2 included people with HIV who had non–central nervous system tuberculosis.

*P* < .05.

CAT-Rapid and MoCA screens were performed in 19 participants with HIV-TBM (the LASER-TBM cohort), and the mean scores (standard deviations) were 16 (3.15) and 21 (3.70), respectively. Seven (34%) and 17 (89%) of the participants, respectively, would have been flagged as having “mild cognitive impairment” on the CATRAPID and MoCA, respectively. A κ coefficient of 0.242 (95% confidence interval, −.179 to .661) for CatRAPID and 0.137 (−.115 to .389) for MoCA equated to “fair” and “slight” agreement, respectively, when comparing these measures with a GDS of ≥0.5 on the cognitive test battery.

Proportionally more participants with HIV-TBM had depression than in comparator group 1 (8 of 34 vs 6 of 66, respectively; *P* = .049); whereas no difference was found between the those with HIV-TBM and comparator group 2 (8 of 34 vs 5 of 26; *P* = .76). In the subgroup of participants with TBM in whom the PAOFI was completed (n = 19) we found that lower cognitive performance was associated with better functional status (mean [standard deviation] PAOFI score in patients with low cognitive performance vs those without, 23.5 [14.8] vs 40.6 [19.1]; *P* = .04). When comparing a cutoff of ≥3 affirmative responses (“functional impairment”), the difference was not statistically significant (5 of 11 [45%] functionally impaired in the group with vs 6 of 8 [75%] in the group without low cognitive performance; *P* = .35).

Given this unexpected finding we explored these cases. Although the PAOFI is a measure of functional status, the questionnaire centers around self-reporting of cognitive symptoms; therefore, low mood, lack of insight, and premorbid status may affect reporting of cognitive functioning. We identified 1 participant whose performance on the cognitive test battery was severely impaired (GDS, 2.85) but whose PAOFI scores were low (affirmative responses, 0; total score, 18), suggesting lack of insight and underreporting of impairment. We identified 2 participants in whom the Beck Depression Inventory suggested clinical depression and whose PAOFI scores were high (ie, high burden of cognitive symptoms) but whose performance on cognitive testing was within normal limits, suggesting overreporting of symptoms associated with low mood. In another participant, PAOFI scores were high (affirmative responses, 12; total score, 71), yet the GDS was within the low-normal range (0.43). However, because this individual had high premorbid functioning (had completed 12 grades of schooling), a drop in cognitive functioning may not have been identified using a GDS cutoff of ≥0.5.

Within the LASER-TBM cohort, 6 of 11 (55%) with low cognitive performance reported missing medication doses, compared to 3 of 8 (38%) with normal cognitive performance. The mean number of missed doses was 2.72 in those with low versus 0.37 in those normal cognitive performance. Neither difference was statistically significant.

## DISCUSSION

Almost half of participants with HIV-TBM demonstrated low performance with cognitive testing. This was significantly more than those among with HIV alone or those with HIV and non-CNS tuberculosis, suggesting that low cognitive performance in HIV-TBM is additional to the CNS effects of HIV and to other mechanisms in non-CNS tuberculosis, such as systemic inflammation and polypharmacy [25, 26].

Low cognitive performance was seen across all cognitive domains in participants with TBM compared with those with HIV only, except for attention and working memory. We did not see cases in which motor/sensory deficits and radiological findings were correlated with a single focal cognitive deficit. These findings characterize low cognitive performance in TBM as generalized, affecting multiple cognitive domains that are, at least in our cohort, beyond what is attributable alone to focal structural deficits. The predominant motor impairment suggests subcortical damage, and sparing of attention and working memory suggests ongoing delirium is unlikely to explain our findings. These observations highlight the limitations of computed tomography in identifying changes such as cortical/subcortical inflammation and microvascular damage, which may present clinically with generalized cognitive deficits. This is important when considering (1) pathogenic mechanisms and (2) suitable imaging techniques for identifying those at risk of impairment.

In the subgroup in which PAOFI was administered, the finding of better self-reported functional status in those with low cognitive performance was unexpected. Assessment of individual cases suggested examples where functioning may have been underreported owing to lack of insight, overreported owing to low mood, or not reflected in the GDS cutoff because of higher premorbid functioning. These examples illustrate the complexity of measuring cognitive and functional performance in diverse populations and the limitations of dichotomized cutoffs for impairment based on cognitive performance alone. Work is needed to explore the association of cognitive performance with clinical indicators of cognitive impairment and functional outcomes and measures of brain injury, an area currently being explored in PWH [23]. Future studies may consider including (1) observer accounts of functional status (eg, Deterioration Cognitive Observee [27]) and (2) more objective functional outcome measures (eg, modified Rankin score).

We found no statistically significant difference in self-reported or objective measures of treatment adherence between those with and those without low cognitive performance, possibly owing to the small number of participants in whom these data were available (n = 19). Medication nonadherence is the major cause of poor outcome in TBM; hence, this potential association should be further explored in larger cohorts as it may provide new avenues to address adherence and improve outcomes in this group.

There are limitations to the current study. First, cognitive test batteries were administered by different individuals across the 3 studies. Although training and alignment were overseen by the same neuropsychologist to minimize interrater variability, subtle differences in administration may have influenced outcomes. Similarly, in 4 domains a single measure was used across all 3 studies and therefore included within the analysis; ideally >1 measure should be used for each domain. This may explain the unexpected finding of preserved attention and working memory despite this being an early indicator of pathology in many subcortical dementias.

Second, there were differences in the timing of investigations related to HIV across the studies; CD4 cell counts were collected at different time points in relation to the cognitive testing, making them not comparable between groups. Given the nature of recruitment (inpatient recruitment for participants with TBM vs outpatient recruitment for PWH), it is likely that HIV disease was better controlled within comparator group 1 than in those with TBM. Of note, however, there was no difference in CD4 cell counts between those with and those without low cognitive performance in the TBM group, and these counts were on average higher in participants with TBM than in those with non-CNS tuberculosis, who performed better on cognitive testing. These, together with differences in cognitive impairment before the development of TBM, could be addressed within a prospective study design, which should be considered within this field.

Third, the lack of TBM case patients with severe (British Medical Research Council grade 3) disease may suggest that the frequency of low cognitive performance is underestimated. Within the LASER-TBM study, recruitment of participants with grade 3 disease was infrequent, and none of the participants followed up with full cognitive testing had grade 3 disease at baseline. Similarly no participants included in the Albertyn study had grade 3 disease. Participants who survive grade 3 disease should be included in future studies to ensure the generalizability of results. Finally, our findings of low cognitive performance 6 months after TBM diagnosis should not be interpreted as a finding of long-term disability in this population. Longer-term follow-up studies are needed to clarify whether cognitive performance improves or worsens in the years following active illness. These studies must incorporate detailed assessment of neurobehavioral functioning, with observer accounts, to elucidate whether low cognitive performance translates to clinically apparent cognitive impairment after TBM. This is timely, given emerging data from ex vivo studies implicating pathogenic mechanisms such as neuroexcitotoxicity leading to neuronal injury [7], mechanisms also described in neurodegenerative conditions [28, 29].

Although the current study provides important characterization of neurological sequalae in TBM, the feasibility of adopting full cognitive testing batteries in TBM-endemic settings is poor. We found only “fair” or “slight” agreement between CatRAPID and MoCA screens and the full cognitive test battery. The MoCA characterized most participants as cognitively impaired and may not be appropriate for this setting; this tool was developed and normed for a North American population, whereas in a low-income periurban South African population one study found that the mean score in cognitively unimpaired, healthy controls was 21.7 or 30 [30]. A receiver operating characteristic analysis is the best method to establish disease-specific cutoffs, but we were underpowered to perform such analysis. Given the high frequency of low cognitive performance within our TBM cohort, a larger study to validate and/or adapt cutoffs for impairment in existing screening tools within the TBM context is required so that potential impairment can be identified where resources are limited. Where resources are available, our results highlight the value of including detailed cognitive and functional assessments as quantitative measures of clinical outcomes within clinical trials in adult TBM.

TBM occurring in people without HIV coinfection may have different cognitive sequalae. TBM has different clinical and neuropathological characteristics in PWH than in those without HIV [31, 32]. In addition, PWH may have underlying HIV-associated brain injury, which could decrease cognitive reserve and increase vulnerability to cognitive impairment from TBM neuropathology. A study of TBM cognitive outcomes in persons without HIV coinfection would provide further insight into the burden of impairment in this context and would be an important comparison for future studies.

In summary, our study demonstrated that low cognitive performance occurred in approximately half of participants of HIV-TBM and is characterized by diffuse impairment affecting multiple cognitive domains. We also demonstrate that low cognitive performance in HIV-TBM is independent of, and additional to, the effects of HIV and non-CNS tuberculosis disease. Future work is now needed to evaluate outcomes at longer-term time points, describe the relationship between cognitive performance, functional status and treatment adherence, and validate sensitive context-specific screening tools to identify individuals at risk, to improve outcomes for patients with TBM.

## Supplementary Data

Supplementary materials are available at *Clinical Infectious Diseases* online. Consisting of data provided by the authors to benefit the reader, the posted materials are not copyedited and are the sole responsibility of the authors, so questions or comments should be addressed to the corresponding author.

## Notes

***Copyright notice.*** For the purposes of Open Access the authors have applied a CC-BY public copyright license to any author-accepted manuscript arising from this submission.

***Financial support.*** This work was supported by the Wellcome Trust (University College London PhD Programme for Clinicians Fellowship [award 175479] to A. G. D.), grant WT097254 to S. M., and grants 104803 and 203135 to R. J. W.); the Francis Crick Institute (support to R. J. W.; the Institute is funded by grant FC0010218 from United Kingdom Research and Innovation (UKRI), Wellcome, and Cancer Research UK (CRUK)); Meningitis Now (R. J. W.); the National Institutes of Health (grants R01AI45436 to R. J. W. and K43TW011421 to S. W.); Cancer Research UK (support to R. J. W.); the UK Medical Research Council (support to R. J. W. and for research reported on the CONNECT cohort [with funds from the UK government’s Newton Fund]); the European and Developing Countries Clinical Trials Partnership (support to R. J. W.); the Discovery Foundation (academic fellowship award to C. A.); and the South African Medical Research Council, with funds received from the South African National Department of Health (support for research reported on the CONNECT cohort ).

## Supplementary Material

ciac831_Supplementary_Data
