# Supplementary material for: Cognitive Impairment in Tuberculous Meningitis
Source: Clin Infect Dis. 2022 Oct 20;76(5):842–9. doi: 10.1093/cid/ciac831 (PMC9989126; doi:10.1093/cid/ciac831)
Supplement: ciac831_Supplementary_Data [file ciac831_supplementary_data.docx]

**Supplementary Material**

**Supplementary Box 1: Cognitive test battery**

| **Domain and measures used** |
| --- |
| **Motor**  Grooved Pegboard: completion time for non-dominant hand  Finger Tapping test: completion time for non-dominant hand |
| **Audioverbal learning and memory**  Hopkins Verbal Learning Test: total learning |
| **Visuospatial learning and memory**  Brief Visuospatial Learning Test-Revised: total learning |
| **Attention and working memory**  WAIS-III Digit span: total score |
| **Processing Speed**  WAIS III Digit Symbol Coding: total score  WAIS III Symbol search: total score  Colour Trails 1: completion time |
| **Executive Function**  Colour Trails II: completion time |
| **Fluency**  Category Word Fluency: total number of animals and total number of fruits and  vegetables named in 1 minute |

**Supplementary Box 2: Comprehensive neurological bedside assessment**

Motor, sensory and cranial nerve examination

Assessment for language deficits, apraxia, visual agnosia, and right hemisphere dysfunction (visuospatial deficit, anosognosia, sensory neglect).

*performed in English with where necessary an isiXhosa translator*
